# The Bidirectional Relationship between Sleep and Immunity against Infections

**DOI:** 10.1155/2015/678164

**Published:** 2015-08-31

**Authors:** Elizabeth G. Ibarra-Coronado, Ana Ma. Pantaleón-Martínez, Javier Velazquéz-Moctezuma, Oscar Prospéro-García, Mónica Méndez-Díaz, Mayra Pérez-Tapia, Lenin Pavón, Jorge Morales-Montor

**Affiliations:** ^1^Departamento de Inmunología, Instituto de Investigaciones Biomédicas, Universidad Nacional Autónoma de México, AP 70228, 04510 México, DF, Mexico; ^2^Area de Neurociencias, Departmento de Biología de la Reproduccion, CBS, Universidad Autonoma Metropolitana-Iztapalapa, Avenida San Rafael Atlixco No. 186, Col Vicentina, Iztapalapa, 09340 Mexico City, DF, Mexico; ^3^Grupo de Neurociencias, Laboratorio de Canabinoides, Departamento de Fisiología, Facultad de Medicina, Universidad Nacional Autónoma de México, México, DF, Mexico; ^4^Departamento de Inmunología, Escuela Nacional de Ciencias Biológicas, IPN Prolongación de Carpio y Plan de Ayala s/n, Col. Sto. Tomás, 11340 México, DF, Mexico; ^5^Unidad de Desarrollo e Investigación en Bioprocesos (UDIBI), Escuela Nacional de Ciencias Biológicas, IPN Prolongación de Carpio y Plan de Ayala s/n, Col. Sto. Tomás, 11340 México, DF, Mexico; ^6^Laboratorio de Psicoinmunología, Dirección de Investigaciones en Neurociencias, Instituto Nacional de Psiquiatría Ramón de la Fuente, 14370 México, DF, Mexico

## Abstract

Sleep is considered an important modulator of the immune response. Thus, a lack of sleep can weaken immunity, increasing organism susceptibility to infection. For instance, shorter sleep durations are associated with a rise in suffering from the common cold. The function of sleep in altering immune responses must be determined to understand how sleep deprivation increases the susceptibility to viral, bacterial, and parasitic infections. There are several explanations for greater susceptibility to infections after reduced sleep, such as impaired mitogenic proliferation of lymphocytes, decreased HLA-DR expression, the upregulation of CD14+, and variations in CD4+ and CD8+ T lymphocytes, which have been observed during partial sleep deprivation. Also, steroid hormones, in addition to regulating sexual behavior, influence sleep. Thus, we hypothesize that sleep and the immune-endocrine system have a bidirectional relationship in governing various physiological processes, including immunity to infections. This review discusses the evidence on the bidirectional effects of the immune response against viral, bacterial, and parasitic infections on sleep patterns and how the lack of sleep affects the immune response against such agents. Because sleep is essential in the maintenance of homeostasis, these situations must be adapted to elicit changes in sleep patterns and other physiological parameters during the immune response to infections to which the organism is continuously exposed.

## 1. Introduction

Sleep is a physiological process that has been proposed to have restorative and regulatory properties [1, 2]. Although it remains unknown what its exact function is, sleep has garnered particular interest in recent years due to its potential influence on the immune system. Many studies have demonstrated that total sleep deprivation and rapid eye movement (REM) sleep deprivation modify various components of the immune system, such as the percentage of cell subpopulations (e.g., CD4+, CD8+, and NK) and cytokine levels (e.g., IFN-g, TNF-a, and IL-1) [3–5]. Also, conversely, sleep patterns are altered during the immune response, suggesting that sleep and the immune response are linked through bidirectional communication.

Sleep can be defined as a state of immobility, resulting from the decreased ability to respond to external stimuli, and is distinguished from coma and analgesia, because it is rapidly reversible. Further, when deprived of sleep, the organism tends to recover, depending of the extent and duration of sleep loss. The existence of this “rebound” after sleep deprivation suggests that sleep is not simply a period in which activity and alertness decline [6, 7]—it is a vital process that modulates various physiological functions.

In mammals and birds, sleep has specific electroencephalographic (EEG) patterns, which divide the sleep process into several stages. In addition, electromyograms (EMGs) and electrooculograms (EOGs) are used to differentiate the phases of sleep. Based on these parameters, several stages of sleep have been proposed: wakefulness, light sleep (2 stages), slow-wave sleep, and rapid eye movement (REM) sleep, each of which has specific electrical patterns [8]. Based on the classification of sleep stages, a hypnogram can be constructed, describing the number of episodes, duration, rhythmicity, and latency of overnight sleep. Sleep patterns differ between species and during ontogeny and are altered in sleeping disorders (dyssomnia) or when a medical, psychiatric, or neurological disease develops [9].

During sleep, important processes occur with regard to endocrine function in mammals, such as rises in the levels of hormones, such as prolactin and growth hormone [10]. On the contrary, cortisol levels decline, peaking before one wakes up [10, 11] which demonstrates the existence of a connection between sleep and other physiological events.

Studies on total sleep deprivation and REM sleep deprivation suggest that sleep has an important function in memory consolidation, learning, and neuronal plasticity [12–14], although it has also been proposed to be a mechanism to conserve and recover energy [1, 15]. Another theory claims that sleep is a process in which functions and cellular components [2, 16] are restored, but some of these studies are controversial; thus, the exact function of sleep remains unknown.

The primary function of the immune system is to defend the body from infections due to pathogens or self-transformed cells through early innate immunity and subsequent adaptive responses.

Innate immunity is the first line of defense. Its two primary functions are to isolate and destroy invading pathogens through inflammatory processes and to recognize and process antigens to affect acquired immunity. Both types of immunity include cellular and biochemical mechanisms that are designed to respond quickly to infections and accurately distinguish between native and foreign materials.

In innate immunity, for example, foreign pathogens are recognized by pattern recognition receptors (PRRs), which are encoded in the germline, have broad specificity for detecting molecular structures that are unique to such organisms, and are evolutionarily conserved. These unique molecular patterns in pathogens are known as pathogen-associated molecular patterns (PAMPs) [17]. PMAPs are generally components of the bacterial cell wall, such as lipopolysaccharide (LPS) and peptidoglycan. Other important PAMPs include *β*-glucan (a cell wall component of fungi) and viral nucleic acids (DNA and RNA), all of which have specific structural characteristics [18].

There are various receptors that recognize PAMPs, the most extensively studied of which are Toll-like receptors (TLRs), comprising 13 types that recognize a wide range of PAMPs. TLRs bind to molecules, such as large lipopeptides in bacteria and mycoplasma [19]. NLRs form another group of TLRs that act as intracellular sensors that detect viral DNA and RNA [20].

The activation of TLRs by their bacterial ligands induces an inflammatory response that stimulates macrophages, which produce proinflammatory cytokines, such as tumor necrosis factor alpha (TNF-*α*), interleukin-1*β* (IL-1*β*), interferon-gamma (IFN-*γ*), and interleukin-6 (IL-6), which coordinate local and systemic inflammatory immune responses. TNF-*α* and IL-1*β* trigger the local endothelium to induce vasodilation and increase permeability of blood vessels, promoting the recruitment of serum proteins and leukocytes to the site of infection. IL-1*β* and IL-6 together, interacting to hepatocytes, activate them to produce acute phase proteins which activate complement and opsonize pathogens, to be phagocytosed by neutrophils and macrophages.

TLRs are expressed in other effector cells of the innate immune system, such as neutrophils, monocytes, NK cells, and *γδ* T cells [18], which can coexpress more than 1 type of TLR. Phagocytic leukocytes, such as eosinophils, basophils, and mast cells, are the principal effectors of innate immunity, the main function of which is to ingest and kill pathogens. Other types of phagocytes participate in these processes, acting as antigen-presenting cells (APCs) and generating antigenic peptides that activate specific immune responses—particularly foreign antigens that are partially degraded by T lymphocytes [21].

Recognition of antigens by the adaptive immune system is mediated by specific receptors. These receptors are also encoded in the germline, and through somatic recombination, random combinations of segments of these genes can generate a large and diverse repertoire of receptors with high specificity [22]. The resulting products are clonally distributed in antigen-specific T and B lymphocytes, which express receptors that are specific for 1 antigen, and specific populations are selected to expand in response to the pathogen [23].

T cells recognize peptides through the T-cell receptor (TCR), which affects disparate mechanisms, depending on the type of T lymphocyte response. There are 2 chief groups of conventional T cells: T helper (Th) cells that express the CD4 coreceptor and cytotoxic T lymphocytes that bear CD8. Both cell types recognize an antigenic peptide that has to be complexed to the major histocompatibility complex class II (MHC II) molecules, whereas B cells recognize the antigen by binding to a 3-dimensional molecular determinant (epitope).

In turn, certain Th cells interact with B cells, through which the latter produces large amounts of immunoglobulin or antibody. Every B cell produces antibodies with a unique specificity that neutralize and destroy the antigen [23].

Innate and acquired immune responses require a network of molecules that signal and orchestrate them. These molecules (cytokines) are synthesized by all classes of immune cells and many other cell types. Generally, cytokines act as proinflammatory, regulatory, or anti-inflammatory molecules and can be classified depending on the subtype of lymphocytes that produced them, as Th0, Th1, Th2, Th3, or Th17, although the actual classification is broader and more complex.

Cytokines participate in innate and adaptive immune responses [24]. Th1 cells and activated macrophages primarily secrete IFN-*γ* and other cytokines that mediate the response against intracellular pathogens and induce B cells to synthesize IgG2 antibodies. Th2 cells preferentially respond to multicellular parasites and produce IL-4, IL-5, and IL-13 [25] which modulate the function of eosinophils, basophils, and mucosal epithelial lymphocytes. IL-5 specifically instructs lymphocytes to produce IgE antibodies. Th17 cells induce cell types, such as epithelial cells, to produce IL-17 and chemokines that recruit neutrophils to the site of infection and are involved in the response against extracellular bacteria and fungi cells [25].

The differentiation of Th cells into various lineages is controlled by master transcription factors, the expression of which is regulated by cytokines that are produced and governed by APCs in response to activation by PAMPs. Thus, the adaptive immune response results in antigen-specific activation that is orchestrated by the innate immune response.

## 2. Effect of Infection on Sleep

The brain is linked to the immune system, and similar interactions occur during sleep, wherein brain activity changes, resulting in the putative “awake brain” and “sleeping brain.” There is evidence that the expression of molecules, such as neurotransmitters, hormones, and cytokines, is modulated while the subject sleeps, and human studies have described changes in the serum levels of some of these components during sleep. Specifically, secretion of IL-1*β*, IL-10, IL-12, and TNF-*α* by monocytes and dendritic cells peaks during sleep, independently of circadian rhythms.

This behavior may be directly related to sleep, because when the animal is deprived of the rhythmicity of these cytokines [26, 27], the changes in expression wane. Also, blood levels of monocytes, T cells, and NK cells follow a clear circadian rhythm regarding the sleep-wake cycle [3]. Notably, other neuroendocrine mediators, such as prolactin, cortisol, and norepinephrine, also exhibit circadian rhythms, but their secretion pattern is more related to the sleep-wake cycle, and all of these compounds modulate the immune response [28].

Conversely, certain cytokines affect sleep, such as IL-1*β*, which, when administered intracerebroventricularly into rabbits and rats, increases the duration of non-REM sleep. This effect is abolished when IL-1*β* antagonists are given [29–31]. Administration of the cytokines TNF-*α* and IFN-*α* has the same effect as IL-1*β* [32–34].

The hypothalamus, hippocampus, and brainstem harbor immunoreactive neurons for IL-1*β* and TNF-*α* and their receptors. These neurons are differentially distributed throughout various regions of the brain, such as the choroid plexus, hippocampus, hypothalamus, and cortex. Notably, these regions might be involved in the regulation of sleep. Further, IL-1*β* has effects on the serotonergic system and governs sleep at several levels [35–37].

This evidence implicates an interaction between components of the immune system and the mechanisms that generate sleep. Thus, the changes that occur during sleep when an immune response is mounted against a pathogen should be described.

Several infectious diseases are associated with sleep disorders. Particularly, infectious agents, such as viruses, bacteria, and parasites, infect the CNS and cause sleep disorders, due to the immune response that is generated against the infection or through direct effects by the pathogen. Moreover, other invasive agents that cause sleep disturbances affect other systems, such as the respiratory and endocrine systems—not those that regulate sleep.

However, most infectious processes, particularly during the acute phase of the immune response, alter sleep patterns, usually protracting the duration of the slow sleep wave (SWS) and consequently decreasing wakefulness and REM sleep. This disruption is commonly observed during an infection and might be a mechanism that is used by an organism to adapt to such circumstances and devote more of its energy to the immune system to clear the infection.

## 3. Viral Infections and Sleep 

Viruses are entities that infect cells, replicating and shedding particles that are called virions. Typically, viruses are composed of DNA or RNA that is covered by a layer of proteins, called the capsid. They differ from other life forms in the cell in that, during replication, they are not covered by the capsid and use the host cell machinery to replicate [38].

Viruses cause many diseases, including pandemics, such as influenza and immunodeficiency virus (HIV). These diseases present with a variety of symptoms, depending on the organ and system that is infected, but many of these conditions are accompanied by sleep disturbances, fatigue, and fever. For example, intranasal inoculation with influenza virus in mice enhances non-rapid eye movement sleep (NREM) and decreases rapid eye movement sleep (REM), despite body temperature declining [39–42].

Genetics also influences the susceptibility and resistance to infectious agents [43–47]. For instance, the increases in NREM sleep during influenza appear to be strain-dependent—C57BL/6 (B6) mice experience greater NREM sleep during infection, whereas BALB/c mice do not [41]. The effects of influenza virus on sleep patterns and specific alterations in sleep have been studied extensively. The well-characterized path and timeline of influenza virus as it travels through the brain suggest that it is a good model for examining the relationship between viral infection and sleep. In 1995, Toth reported that, in addition to the differences in alterations in sleep patterns between strains due to influenza infection, slow wave sleep increases in C57BL/6 mice—a typical alteration in the circadian rhythm of sleep in rodents, depending on the strain [41].

Lethargic encephalitis (LE) has been described for over 1 century, but little is known about its relationship with depression. Instead, LE has been attributed to the immune response against the central nervous system. Despite being characterized by Hippocrates and Sydenham in the 19th century, its etiological agent has not been identified. LE is a central nervous system (CNS) disorder, in which pharyngitis and, subsequently, sleep disorders, including primary hypersomnolence, and various forms of ophthalmoplegia develop in the early phase [48].

LE was named in 1916 by Constantine von Economo, who observed that, in addition to the remarkable sleep disorders, lethargy, and extrapyramidal movements, surviving patients developed neuropsychiatric disorders, such as catatonia, obsessive-compulsive disorder, and mutism [49]. A hallmark of LE is atrophy of the encephalitis basal ganglia and midbrain structures. Since the 1916–1927 epidemic, few cases of LE have been reported. The LE epidemic occurred during the influenza pandemic of 1918, linking the 2 outbreaks [50]. A recent examination of brain samples with LE demonstrated a lack of viral RNA [51], and other studies in patients with the same clinical features indicate that LE correlates more to an autoimmune disease—95% of subjects had antibodies against antigens of their basal ganglia.

Poliovirus is another viral infection that affects the CNS and causes sleep disorders. Patients who are infected with this virus and experience an acute infection develop a syndrome several years later, including neuromuscular and respiratory symptoms, and comorbid sleep disorders, characterized primarily by obstructive apnea and hypopnea, which affects oxygen desaturation during sleep [52, 53]. Other studies have shown that, in addition to difficulties of swallowing and breathing, periodic limb movements occur during sleep, which might be attributed to dysfunction of motor neurons in the brainstem, probably due to abnormal production of dopamine, which is involved in respiratory disorders that are associated with sleep [54].

Similarly, infection by human immunodeficiency virus (HIV) affects the CNS. Considered the most significant pandemic of our time, patients who are infected with this lentivirus experience fatigue and sleep disorders in the asymptomatic stage [55]. Also, HIV impairs reasoning and memory [56]. As HIV infection progresses, patients can present with psychiatric and cognitive dysfunction, which might be part of the well-known neuropathology.

Early polysomnographic studies of HIV-infected patients have reported altered sleep organization that occurs in the early stage of and throughout infection in adults and children who have more wake time after sleep onset [57]. In these patients, REM sleep declines, SWS is present, and sleep spindle- and K-complex densities are reduced. These symptoms progress, depending on the disease, and ultimately, total sleep time and SWS decrease and wakefulness increases [58].

Other studies in HIV-positive, but asymptomatic, patients have described conflicting data. Whereas Norman et al. reported an increase in the percentage of SWS, the number of periods of stage 1 sleep and REM and the number of awakenings [59], normal periods in SWS, and REM sleep have been observed by others [60]. Nevertheless, both studies noted greater sleep latency and lower percentage of stage 2 sleep [59, 60]. Norman et al. ruled out the possibility that the alterations were due to the medication, anxiety disorders, or depression, suggesting instead that they were attributed to mobilization the immune response against the central nervous system [59]. Subsequently, it was reported that, in asymptomatic patients, periods of wakefulness, SWS, and REM sleep were dispersed uniformly throughout the night [59]. Further, other changes occurred in the microstructure of sleep in asymptomatic patients, in whom the cyclic alternating pattern occurred at a significantly higher rate, unrelated to psychiatric or neurological disorders [61].

Because sleep disturbances appear at an early stage of infection, before the disease has developed significantly, it has been proposed that these alterations are caused by direct infection of the CNS and involve certain viral peptides [62], such as components of the capsid. Four hours after intraventricular administration of the gp120 glycoprotein (part of the capsid protein), in rats, alterations in sleep patterns that are similar to those that occur during HIV infection in humans are seen [63]. Furthermore, injection of gp120 decreases glucose utilization in the suprachiasmatic nucleus and lateral habenula [64]. Further, prolonged administration in rats of other glycoproteins, such as gp160 and gp41—also components of the envelope—increases REM sleep and alters sleep fragmentation and the low-frequency components in the EEG. However, if they are administered continuously, they induce mild febrile responses [65].

Other studies have reported that feline immunodeficiency virus- (FIV-) infected cats presented with injury and infiltration of mononuclear cells into various areas of the CNS, (such as the glial nodules during hippocampal rotation and the cortical and subcortical regions). Other structures in which the virus was detected are cortex, midbrain, and cerebellum. The virus location is accompanied by slowdown of the predominant frequency in the electroencephalogram (EEG) and alterations in sleep architecture, including fragmentation and displacement of the SWS [66]. Infected cats spend 50% more time awake, and experience more transition periods between wakefulness and sleep, and a 30% reduction in REM sleep and less frequencies of sleep spindles are observed during the SWS.

Many of these changes in infected cats approximate to those in persons infected with HIV or that develop AIDS [67]. Thus, this model is a good experimental approach to examine the mechanisms by which HIV infection induces changes in sleep patterns, possibly through direct action of viral components in the CNS or by the immune response to the virus or its proteins during infection. Other viruses also directly affect the CNS, such as rabies. Rabies-induced alterations begin in the early stages of infection and disappear at the same time that the clinical signs wane. Rabies does not affect any significant EEG abnormalities in mice but causes sleep disturbances. The hallmarks of this disorder are fewer REM sleep episodes and greater periods of wakefulness. Compared with fixed rabies virus, street rabies alters EEG recordings, and sleep and waking stages are replaced by a pathological sleep stage.

To determine the function of immune responses in these disorders, infected immunosuppressed mice were studied to note the changes in sleep patterns versus immunocompetent mice. By EEG, both groups experienced the same alterations; thus, they are likely to be caused directly by pathogenic mechanisms of the virus rather than the immune responses against it [68].

Varicella zoster a neurotropic virus also causes sleep disturbances; patients develop fatigue, hypotension, and sleep disorders [69]. Hepatitis C virus affects psychiatric disorders and sleep disturbances, but whether it causes disease in the CND is unknown. Approximately 60% of patients with chronic hepatitis C develop psychiatric disorders, including sleep disturbances, which also manifest during therapy with interferon-*α* (IFN-*α*) [70]. Hepatitis C patients have a high incidence of sleep disorders, depression, and anxiety during and after treatment with IFN-*α* and without treatment; thus, the effects on sleep appear to depend on the infection [71].

On the contrary, Raison showed that IFN-*α* therapy exacerbates these disorders—such patients had a significant decrease in sleep time in stages 3 and 4 and in sleep efficiency (total sleep time/time spent in bed × 100), whereas they experienced increased fatigue, took fewer naps during the day, and had greater plasma cortisol levels [72]. These data are indicators of stress in such patients and suggest that sleep disorders, depression, and anxiety result from deterioration of the emotional state and the immune response against the virus.

Similarly, another study reported that hepatitis B-infected individuals who also had sleep disorders and were treated with alternative medicines (extracts of Jujube seed, Anemarrhena rhizome, Poria sclerotium, and Ligusticim wallichii rhizome), not IFN-*α*, improved their sleep quality. Sleep I and sleep II improved, whereas sleep III, sleep IV, and REM sleep increased significantly [73]. This evidence supports the finding that sleep disorders occur in patients who have not been given IFN-*α* and suggests that they are caused by altered levels of this cytokine, indicating that continued exposure to cytokines and innate immune molecules, such as IFN-*α*, reduce sleep continuity and induce a pattern that is consistent with insomnia and arousal.

The various alterations in the patients with hepatitis C also occur during influenza virus infection, which decreases the amplitude of delta waves during SWS sleep in mice and induces hypothermia and decreases locomotor activity clinically. Other strain-dependent alterations in mouse have been observed; for example, infected B57BL/6 mice spend more time in SWS during the dark period, resulting in loss of the circadian rhythm of sleep 4 days after infection. Further, total sleep time rises after viral challenge in immunized mice. On the contrary, BALB/c mice did not develop any of these disorders [41], implicating genetic differences and disparities in the type of immune response between strains with regard to the effects of infection on sleep patterns. This behavior has been attributed to the MC10-12 region of chromosome 6, which is related to prostaglandin metabolism.

As in C57BL/6 mice, Swiss-Webster mice differed in their response on infection with a lethal (H1N1) and nonlethal strain (H3N2). H1N1-infected young and adult mice had lower body temperature and locomotor activity and greater SWS, whereas REM sleep was suppressed. However, compared with the H3N1 virus, the sleep disturbances due to H3N2 virus were less extensive, despite being administered at a 10-fold higher dose [40]. Thus, the changes that are caused by the virus are mouse strain-dependent and are perhaps due to differences in the immune response against each viral strain.

Sleep disturbances might be part of the clinical profile that develops to infection. In a study of men and women who were infected with type 23 rhinovirus, the patients had decreased sleep time, whereas sleep efficiency fell by 5% during the active period of the virus, compared with its incubation period. These changes were greater in asymptomatic individuals and thus might be related to the acuteness of the immune response against this virus, at least in this infection [74].

**Figure 1:**
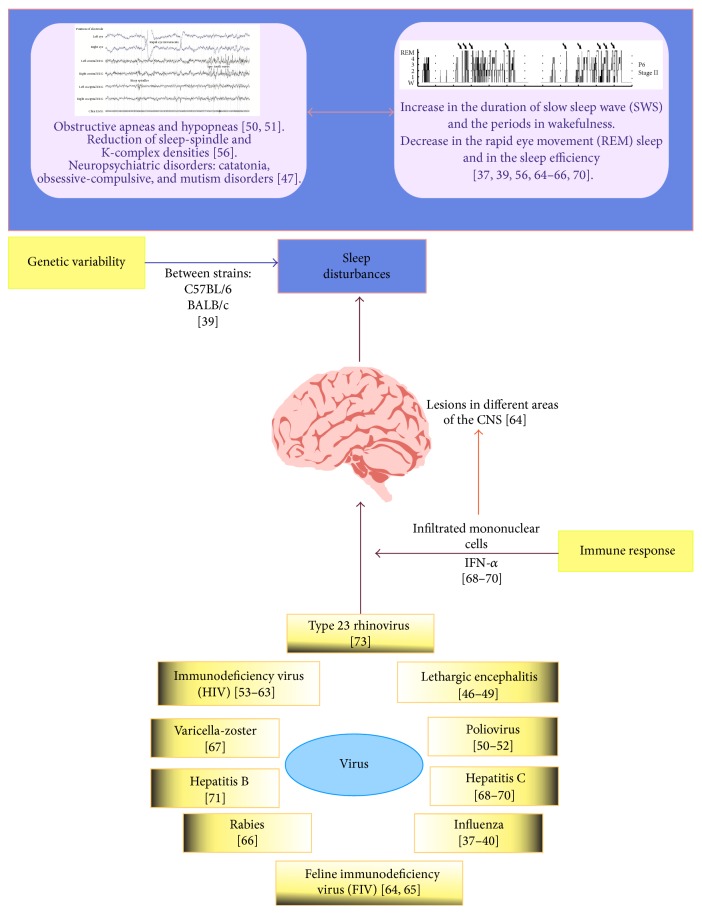
Connectivity between viral infections and sleep. Sleep disorders that are caused by parasites include increased duration of slow sleep wave (SWS), periods of wakefulness, and a decrease in rapid eye movement sleep (REM), as well as, in general, sleep efficiency. Other disorders comprise alterations in electroencephalographic characteristics, such as reductions in sleep-spindle and K-complex densities. These changes may be caused by activation of the immune system and the consequent production of cytokines that have various effects on CNS structures, modulating the form and quantity of sleep. Such pathogens can disrupt sleep indirectly, causing respiratory dysfunction, obsessive-compulsive disorder, and mutism, all leading to sleep disturbances, such as sleep apnea-hypopnea syndrome, that accompany psychiatric disorders.

Despite these studies, the alterations that are caused by viral infections are not well defined—whereas some promote various stages of sleep, others delete or disrupt its rhythm. These effects might be mediated by direct mechanisms of the virus against CNS or the type of immune response that is generated to contend with these infections, which remain unknown. In Figure 1, a chart of the connectivity between viral infections and sleep is depicted.

## 4. Bacterial Infections and Sleep 

Bacteria are another large group of pathogens that infect and cause disease, significantly altering overall functioning of the body. Specifically, certain molecules from bacteria induce sleep, such as components of the cell wall. For example, muramyl peptide lengthens the SWS in rabbits, rats, and dogs [75]. However, intracerebroventricular and intravenous administration of LPS increase the SWS and its amplitude and suppress REM sleep in rabbits [76].

A study in which humans were inoculated with Salmonella abortus endotoxin reported significant declines in waking and REM sleep, which were accompanied by greater non-REM sleep [77]. In contrast to the changes in experimental animals, this endotoxin did not increase the amplitude of delta waves [77]. A subsequent study demonstrated that Salmonella abortus endotoxin reduced the total duration of NREM sleep, whereas waking and sleep latency and daytime sleepiness increased [78].

Toth and Kreuger (1988) [79] reported that sleep is altered during the course of an infection—not solely by inoculation with antigens. In their study, electrodes were implanted in rabbits, which were then inoculated intravenously with* Staphylococcus aureus*. With regard to changes in sleep patterns, the total SWS, amplitude, and duration of episodes rose. 10 hours after inoculation, REM sleep was suppressed and remained at low levels for the next 38 h. The effects of infection on sleep were attenuated by antibiotics (cephalothin). The same alterations as with cephalothin were observed when dead bacteria were used to inoculate the animals [79].

A subsequent study showed that infection of rabbits with* Escherichia coli*,* Candida albicans*, and* Pasteurella multocida* had affects similar to* S. aureus* infection [80, 81]. On the contrary in a separate study, bacterial colonization in rats did not affect the sleep patterns in the first week after infection, although the number of episodes, frequency, and percentage of REM sleep declined in the second week. These results indicate that noninvasive bacterial colonization decreases REM sleep without causing fever, rendering it a sensitive indicator of the degree of bacterial colonization [82].

Clinical data show that bacterial infections also affect sleep disturbances. In contrast to the alterations in experimental animals, human Lyme disease, caused by the bacterium* Borrelia burgdorferi*, is associated with chronic fatigue and sleep disorders, including insomnia. Greenberg et al. reported that the chief sleep disorders that are cited are difficulty falling asleep, frequent nocturnal awakenings, excessive daytime sleepiness, and restless leg syndrome. To a lesser extent, increased sleep latency, decreased sleep efficiency, a higher arousal index, and sleep fragmentation are observed in these patients [83]. Notably, alpha waves (regular fusiform patterns that are usually present in sleepy or relaxed individuals) were also observed in the stages of non-REM sleep. These changes constitute the clinical presentation of Lyme disease [83], although the underlying mechanisms remain unknown.* Bordetella pertussis* induces alterations during sleep, such as epileptic seizures, choking sounds, and sleep walking. Such parasomnias are linked to the infection and disappear on clearance of the pathogen [84].

In contrast to these infections, sleep disorders develop during or after bacterial infection of the respiratory system. However, it has been argued that such sleep disorders are attributed to the effects of the infection on the respiratory system. Bacterial meningitis and rhombencephalitis caused by* Listeria*, sarcoidosis, and pneumococcal meningitis induce sleep disturbances. During sarcoidosis, general apnea develops, and the alterations in* Listeria *infections are likely to be elicited by lesions in the reticular formation, disrupting the respiratory rate and causing oxygen desaturation in REM and non-REM sleep [85–87]. Under these conditions, many arousals, and the quality of sleep declines. Patients with* Listeria* infections have increased total amounts of sleep, characterized by greater daytime sleep; less nighttime sleep; and longer durations of observed awakenings during the night, resulting in alterations in the sleep-wake cycle—the hallmark symptom in these patients. Yet, the mechanisms of these changes remain undetermined.

Other sleep disorders, such as narcolepsy, might also be related to bacterial infections. A large proportion of patients with narcolepsy have antibodies to* Streptococcus* (anti-streptolysin or ASO) and* Helicobacter pylori* (anti-Hp Ig)—2 bacterial infections that are associated with autoimmune diseases [88] and might trigger narcolepsy through autoimmune mechanisms.

**Figure 2:**
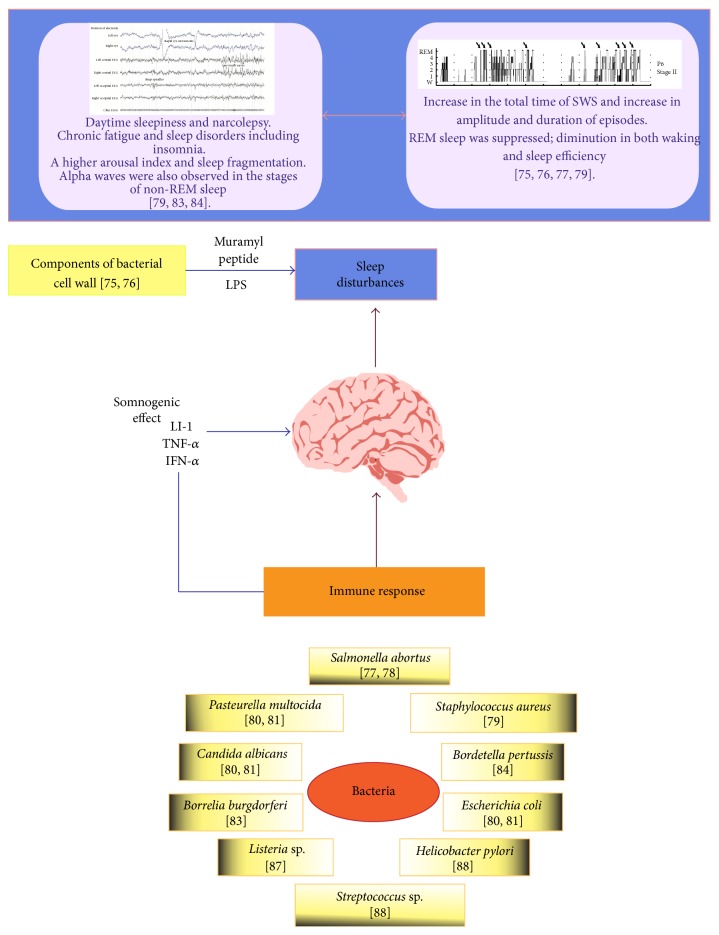
Effect of bacterial infections on the sleep process. The sleep disorders that are caused by bacteria include daytime sleepiness and narcolepsy, chronic fatigue, and insomnia, which are accompanied by higher arousal index and sleep fragmentation. Bacterial infections also cause other alterations, such as increases in the duration of slow sleep wave (SWS) and periods of wakefulness. Also, rapid eye movement (REM) sleep and sleep efficiency decrease in bacterial infections. Wall components of bacteria (primarily LPS) are strong inducers of proinflammatory cytokines, which is one possible mechanism by which the infection causes sleep disorders.

As with viruses, bacterial infections cause various sleep disturbances, depending on the area of the brain that is affected. However, no direct infection of the CNS changes wakefulness or SWS, possibly due to the influence of certain components of the immune or inflammatory response, such as prostaglandin D2, the synthesis and release of which are promoted by bacterial infections.  Figure 2 depicts a chart of the complex effects of bacterial on sleep patterns.

## 5. Parasitic Infections and Sleep 

Parasitic infections can also alter sleep patterns, due to the resulting immune response or through direct effects. Because parasites are multicellular organisms, they can modify certain behaviors to facilitate infection and complete their life cycle [89]. It is possible that sleep is a state in which they can exploit the infected host, because the responsiveness to external stimuli declines, rendering the host more susceptible to infection. However, there is little evidence of this relationship.

As discussed, sleep is a physiological state that it is linked to the maintenance of homeostasis. To achieve this goal, cerebral mechanisms that regulate sleep have a reciprocal relationship with the endocrine and immune systems. Acute and chronic sleep deprivation modifies the immune response, and conversely, immunological challenges alter normal sleep patterns. Thus, sleep impairments are expected to facilitate parasitic infection.

Nevertheless, this issue has not been studied extensively. Human African trypanosomiasis (HAT) is the most, well-characterized parasitic infection that affects sleep patterns. HAT is associated with severe disturbances in nervous system functions, such as endocrine and neuropsychiatric symptoms, sensory alterations, and dramatic changes in sleep patterns. These changes manifest as the complete loss of circadian rhythm, an increase in total sleep time, and narcoleptic-like episodes, defined as direct transition from wakefulness to REM sleep. Based on the changes in sleep patterns, HAT is also designated sleeping sickness [90].

Two subspecies of the hemoflagellate* Trypanosoma brucei*, transmitted by populations of the tsetse flies in sub-Saharan Africa, cause HAT.* Trypanosoma brucei* gambiense causes the West African, or Gambian, form of the disease, and its main host is human. The disease progresses slowly over months or years, increasing the likelihood of parasitic transmission to the tsetse fly vector.* Trypanosoma brucei* rhodesiense causes the East African, or Rhodesian, form of sleeping sickness, and its main host is cattle; when humans are infected, the course of the disease accelerates, developing within several weeks to months [91].

The hallmark alterations in HAT patients are as follows: (i) polyphasic sleep-wake patterns, leading to the profound disruption in the alternation between sleep and wake times over 24 h and (ii) changes in the internal structure of sleep, characterized by slow rapid eye movement (SOREM) episodes, which can appear earlier than the sleep-wake alterations [90]. Uncharacteristic morphological alterations are also observed in the electroencephalographic (EEG) records of HAT patients. The loss of circadian rhythm affects other endocrine rhythms; for example, the release patterns of prolactin and growth hormone are disrupted. The severity of the dysregulation of the sleep-wake cycle, with short sleep episodes, is nearly equal between day and night and is proportional to that of the disease. These findings indicate that sleep-wake changes in HAT are not directly related to the presence of the parasite in the brain and implicate slow, reversible functional alterations and compensatory mechanisms [92]. Animal models of HAT have been developed to understand the pathogenic mechanisms that lead to immune system alterations. In a study in which rats were infected with* Trypanosoma brucei brucei* analysis of the spontaneous sleep-wake architecture demonstrated an increased proportion of slow-wave sleep (SWS) and less wakefulness 2 days before death. Considerable sleep fragmentation was observed in infected rats, which experienced numerous changes in sleep-wake stages and more episodes of wakefulness and SWS. Infected rats developed a fragmented pattern of SWS and had reductions in mean paradoxical sleep (PS) latency, disrupting the PS-SWS sequences. Abnormal transitions, particularly the appearance of sleep-onset REM (SOREM) episodes, marked the dysregulation of the internal sleep structure [93].

Other parasitic infections can cause sleep disturbances, such as Plasmodium. The symptoms of malaria during acute infection are periodic fever with shivering, headache, body ache, sleepiness, and loss of appetite, which are caused by proliferating malaria parasite cells in the erythrocytic stage. Recent evidence suggests that these effects are directly related to the finding that* P. falciparum* synthesizes PGD2, PGE2, and PGF2a disparately from PG biosynthesis by mammalian cyclooxygenase. PGD2 is a somnogenic substance, suggesting that malaria parasites have certain strategies to evade host defenses and establish infection [94].

The neurological manifestations in filariasis have received little attention, due to the frequency with which this condition is superimposed on other diseases with meningovascular involvement, to which fatal complications have been attributed. In 1950, Kenney and Hewitt identified the psychoneurotic manifestations as loiasis, insomnia, headache, nervous depression, and abnormal irritability [95]. The first concerned a man aged 28 years who developed a change in personality that was accompanied by torpor. These symptoms were sufficiently severe for him to return to Europe. During his leave, he suffered from headache again with acute mental symptoms and eosinophilia, ranging from 50% to 56%. The removal of a worm (*M. boa*) from the eye ameliorated all his symptoms and decreased eosinophilia to 3% [96].

There are reports that other parasitic infections cause sleep disorders in the host, primarily due to the clinical features of these parasitemias. Hookworm-related cutaneous larva migrans (CLM) is a parasitic skin disease that is caused by the infestation of human skin by larval nematodes, such as* Ancylostoma braziliense, Ancylostoma caninum,* or* Uncinaria stenocephala*. These nematodes usually parasitize dogs and cats [97]. Because humans are an incidental host, in whom normal larval development is impaired, CLM is self-limiting. However, creeping eruption can persist for several months [98]. Sleep disturbances in CLM are frequent. Patients report delayed sleep onset and sleep fragmentation due to frequent awakening episodes, all of whom attribute this complaint to itching—84% of patients complain about sleep disturbances. Although sleep disorders have many causes, we speculate that, in patients in the above-mentioned study, altered sleep results from severe pruritus that is caused by the lesions [99]. Over 40 years ago, several groups performed studies on onchocerciasis, noting that the prevalence of epilepsy was higher in infected patients, which prompted them to consider that there was a causal relationship between the conditions [100]. All seizure types can be activated by sleep deprivation—a phenomenon that is reported in patients of all ages, although it occurs more frequently in younger subjects [101].

In the cases of parasitic infections that we have discussed in this section, the mechanisms by which they affect sleep remain unknown. Infections, such as malaria, filariasis, and trypanosomiasis, appear to change sleep patterns by modulating the immune response. To this end, our group has observed that experimental infection of rats caused with* Trichinella spiralis* significantly increases NREM sleep after 15 days. This infection comprises a local phase in the intestinal epithelium, lasting up to 5 days after infection, and a systemic phase. At 15 days of infection, the parasite is considered to be present systemically. During this period, a local immune response develops that is characterized by fewer total T cells and more B cells and gamma-delta cells in mesenteric lymph nodes, and there are an inflammatory infiltrate and eosinophilia in the intestinal epithelium (unpublished).

The importance of sleep in immune system function has been examined by many groups. In 2009, Preston and colleagues studied the possibility that sleep evolved across species to afford the organism special protection against parasitic infections. They also analyzed the correlation between sleep duration and parasitic infection levels in 12 mammalian species, finding a negative correlation.

**Figure 3:**
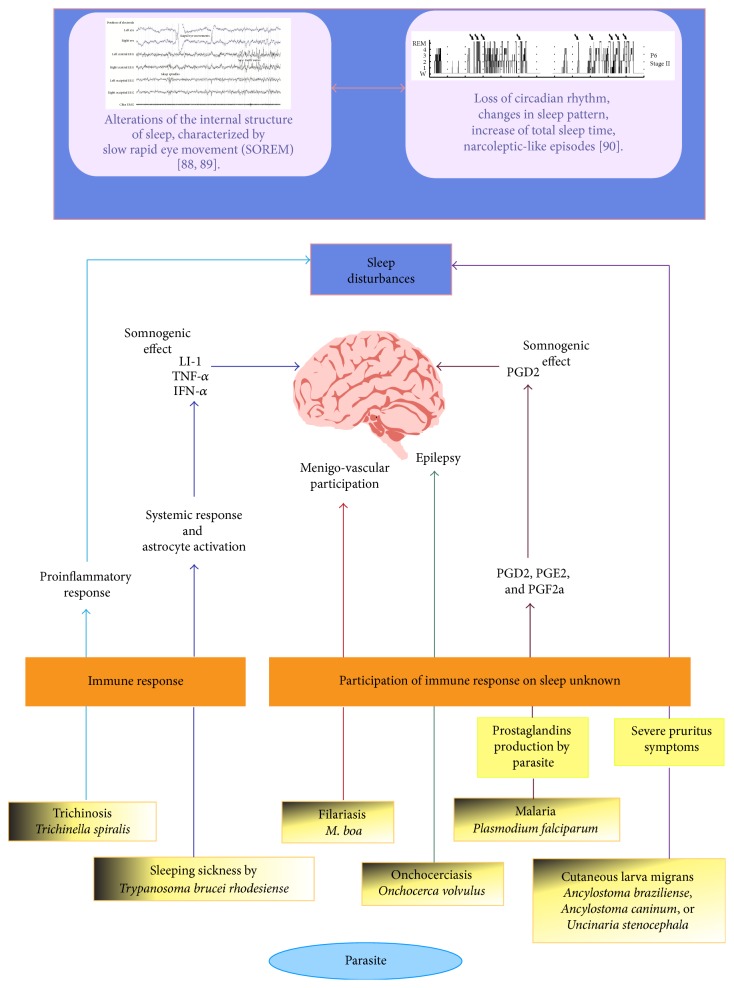
Relationship between sleep and parasitic infections. The sleep disorders that are caused by parasites include changes in sleep patterns, such as the amount of total sleep, and in the duration of each stage (wakefulness, sleep stages 1 and 2, slow wave sleep, and REM sleep). Other disorders comprise alterations in the sleep-wake transition at sleep onset and between sleep stages and electroencephalographic disorders. The disorders in parasitic infections, such as trypanosomiasis and trichinosis, have an immune component. The prostaglandins PGD2, PGE2, and PGF2a are induced by these infections; PGDs are somnogenic substances, explaining the effects of these parasites on sleep. The disorders in sleep due to parasites can modify certain behaviors to facilitate parasitic infection and completion of the life cycle.

A longer duration of sleep correlated significantly with lower levels of parasitic infection. Thus, the authors concluded that sleep evolved to protect animals from parasitic infections [102]. The possible relationship between sleep and parasite infections is depicted in Figure 3.

## 6. Concluding Remarks

In recent decades, much experimental and clinical evidence has been generated on the existence of and interaction between the neuroendocrine and immune systems. This communication network allows the body to maintain homeostasis, particularly when it is required to respond to a stimulus, such as an infection. During an infection, the body must alter many of its metabolic functions to eliminate the pathogen. For example, it must devote most of its energy to the immune system, leaving the remainder of the body with less input.

However, the mechanisms of these changes have not been determined entirely, particularly the brain mechanisms of sleep and the immune response. In general, these processes are linked, based on the effects of immune modulators (cytokines) and the sleep mechanisms and the resulting changes in the sleep-wake cycle and the effect of neurotransmitters in modulating sleep during an immune response. Certain cytokines influence sleep mechanisms, such as IL-1*β*, which when administered intravenously or intraventricularly in rabbits induces a 60% to 70% increase in the amount of NREM sleep time. The administration of TNF-*α* and IFN-*α* has the same effect, which might be mediated by IL-1. IL-1 receptors are expressed in various structures of the brain, and there are immunoreactive hypothalamic neurons to IL-1, which, when coupled with the effects of IL-1 on the serotonergic system, could explain how cytokines facilitate sleep.

Further evidence supports the hypothesis that sleep modulates immune system cells and modulators; women who were deprived of sleep for 77 hours under battlefield-like conditions experienced changes in IFN production and phagocytic activity. Subsequent studies reported that sleep deprivation decreases lymphocyte blastogenesis and NK cell activity and upregulates IL-1 and IL-2. Further, youth who have been sleep-deprived for 64 h experience a significant rise in NK cells, granulocytes, and monocytes. Other studies in rats using a strategy that was designed to perform selective deprivation of REM sleep have reported increased total systemic leukocyte and IgM levels at 96 hours of deprivation. Changes were also observed at 72 hours of deprivation, including enhanced plasma levels of IL-1*α*, IL1-*β*, IL-6, IL-10, TNF, and IL-17A. These findings suggest that deprivation of total sleep and REM sleep entails modulation of the immune system, increasing the inflammatory processes or favoring a certain cellular response.

Conversely, the immune response effects alterations in sleep patterns. Modulators and immune system innate components might be involved in the mechanisms of the changes in sleep patterns. Based on these interactions, the relationship between these processes (sleep and the immune response) is critical in maintaining homeostasis and fending off parasites.

With regard to this hypothesis, Preston and colleagues recently examined the possibility that sleep helps protect the body against parasitic infections. In 12 species of mammals, they demonstrated that sleep duration correlated negatively with the level parasitic infection, prompting the authors to conclude that sleep evolved to protect animals from parasitic infections.

**Figure 4:**
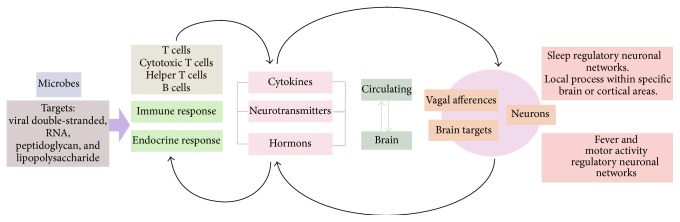
Bidirectional interactions between immunity to infections and sleep. The immune response to the invasion of a pathogen and the consequent secretion of immunological mediators, such as interleukins and cytokines, are accompanied by responses by the endocrine and nervous systems, such as the secretion of cortisol and epinephrine. These substances can cross the blood-brain barrier to reach their receptors in various neural structures or may have a vagal input to modulate the responses that maintain homeostasis. This modulation can also be exploited by pathogens to ensure establishment of the infection and completion of its life cycle. However, this series of events has a complex relationship: infections can modulate patterns of behavior, such as sleep, and such primary functions can alter immune and endocrine system functions. For example, the effects of sleep deprivation on the immune and endocrine response support that sleep is fundamental in maintaining homeostasis—its absence leads to physiological disorders and possibly death. Thus, complex systems must be studied to identify the interactions between 2 or more variables in various contexts to determine the mechanisms that are involved in preserving this balance.

We are merely beginning to understand how infections change sleep patterns, and why sleep is altered during illness remains unknown. One hypothesis suggests that altered sleep during infection is a component of the acute phase response, promoting recovery during illness, likely through mechanisms that involve cytokines and their receptors, as well as receptors of the innate immune system (Figure 4).
